# Role of Flotillin-1 In *Listeria monocytogenes* Cell-to-Cell Spreading

**DOI:** 10.1093/infdis/jiaf452

**Published:** 2025-08-26

**Authors:** Petra A McLeod, Julian A Guttman

**Affiliations:** Department of Biological Sciences, Centre for Cell Biology, Development, and Disease (C2D2), Simon Fraser University, Burnaby, British Columbia, Canada; Department of Biological Sciences, Centre for Cell Biology, Development, and Disease (C2D2), Simon Fraser University, Burnaby, British Columbia, Canada

**Keywords:** endocytosis, bacterial spread, host-pathogen interactions, *L. monocytogenes*, reggie-2

## Abstract

*Listeria monocytogenes* spreads intercellularly by creating actin-rich projections that are endocytosed into recipient cells. Caveolin-mediated endocytosis has been implicated in this process, accounting for about 70% in cell-to-cell spread in cells depleted of caveolin-1. Thus, additional mechanisms may contribute to the remaining spread, and we examined the role of flotillin-based endocytosis. We found that flotillin-1 localized to *L. monocytogenes* invaginations in recipient cells and depletion of flotillin-1 significantly impaired bacterial transfer. Similarly, preventing endogenous flotillin-1 from membrane association significantly reduced bacterial spread. To evaluate whether there was interplay between flotillin-1– and caveolin-1–mediated endocytosis at *L. monocytogenes* invagination sites, we measured the area of spread in cells with both caveolin-1 and flotillin-1 knockdown and found a further significant decrease in spread and many cells with complete blockage. This work demonstrates that flotillin-based endocytosis is crucial for cell-to-cell spreading of *L. monocytogenes* and that this endocytic strategy can internalize large membrane protrusions.

*Listeria monocytogenes*, the causative agent of listeriosis, has a death rate of ~20% in people that it infects [1]. Following ingestion, these foodborne bacteria enter enterocytes, then disseminate throughout the body, infecting the gastrointestinal system, blood, as well as the brain [2]. These microbes infect pregnant women at higher rates than the rest of the population and once pregnant women are infected, *L. monocytogenes* can infect the placenta, breach the placental barrier, and infect the fetus [2].

*L. monocytogenes* initially infect cells by using internalin ligands, which exploit clathrin-mediated endocytosis to enter the host cells [3–6]. *Listeria*-containing vacuoles formed in this process are rapidly lysed through the actions of listeriolysin O and phospholipases, enabling the bacteria to live in the cytosol, where they replicate and ultimately recruit actin filaments, which form comet/rocket tails that are used for their intracellular motility [7]. Individual bacteria, propelled by separate comet tails, eventually reach the plasma membrane, where the propulsive forces against the plasma membrane drive the creation of actin-rich protrusions (or listeriopods), which are led by each bacterium and form corresponding invaginations in the neighboring cells [8–14]. These invaginations have been shown to use caveolin-1–mediated endocytosis to internalize the entire listeriopod into the adjacent cell [15–17]. However, in cells depleted of caveolin-1, cell-to-cell spreading was not eliminated, as 30% remained.

It has been established that clathrin is not recruited to sites of *L. monocytogenes* cell-to-cell spreading [16]. Thus, to evaluate the potential of other endocytic mechanisms being involved in these bacterial spreading events, we explored flotillin-1–mediated endocytosis. Flotillin-1, also known as reggie-2, is a membrane-associated protein that is recruited to plasma membrane through palmitoylation. Flotillin-1 is part of the SPFH (stomatin, prohibitin, flotillin, and HflC/k) superfamily, is expressed in all cell types and is involved in clathrin-independent endocytosis of various proteins [18–25]. We found that flotillin-1 recruited to *L. monocytogenes* generated invaginations. This recruitment caused the internalization of *L. monocytogenes* in adjacent cells and required flotillin-1 palmitoylation. Finally, we demonstrated that the combined depletion of caveolin-1, together with flotillin-1 in receiving cells, could block cell-to-cell spreading of *L. monocytogenes*.

## METHODS

### Antibodies, Reagents, DNA Constructs, and Small Interfering RNA

The following antibodies and reagents were used in this study: Alexa Fluor 594–conjugated phalloidin (Invitrogen), CellTracker Blue (Invitrogen), Alexa Fluor 488 goat anti-rabbit antibodies (1 µg/mL), mouse anti–α-tubulin (Developmental Studies Hybridoma Bank 12G10, 1:1000), horseradish peroxidase–conjugated goat anti-rabbit/mouse antibodies (1 µg/mL), rabbit anti–flotillin-1 (2 µg/mL) (Biorbyt), and rabbit anti­–flotillin-2 (1:500 for Western blot) (Sigma-Aldrich). DNA constructs used in this study included green fluorescent protein (GFP)–flotillin-1 (Origene), tetracycline reponse element-enchanced green fluorescent protein (TRE-eGFP)-F1C and TRE-eGFP-F1A (gifts from Linda DeGraffenried; Addgene plasmids 211457 and 211458), and mCherry–caveolin-1 (gift from Michael Davidson; Addgene plasmid 55008). A smart pool of flotillin-1 targeting small interfering RNA (siRNA) and control siRNA scramble was purchased from Dharmacon (Horizon Discovery).

### Cell Culture

Jeg3 and HeLa cells were purchased from the American Type Culture Collection. HeLa caveolin-1 knockdown (KD) cells were used as described elsewhere [16]. Jeg3 and HeLa/HeLa caveolin-1 KD cells were cultured using minimum essential medium (MEM) and Dulbecco modified Eagle medium (DMEM) supplemented with 10% fetal bovine serum (FBS) (Gibco). HeLa caveolin-1 KD cells were selected for with puromycin (1 µg/mL), as described elsewhere [16]. All cell lines were maintained with 5% carbon dioxide at 37°C. To seed for experiments, the cells were washed 3 times with Dulbecco's phosphate-buffered saline (PBS) without calcium ion or magnesium ion (PBS^−/−^) (Gibco) and trypinized with 0.05% trypsin–ethylenediaminetetraacetic acid (EDTA) (Gibco). Trypsin was neutralized with medium containing 10% FBS. The cells were gathered in a Falcon tube and pelleted at 1000*g*. After resuspension in medium containing FBS, the cells were counted and seeded into 6-well or 24-well clear polystyrene plates at the appropriate density, with or without coverslips.

### Transfections

DNA transfections were performed using JetPRIME (Polyplus Transfection) according to the manufacturer's instructions by combining 1.5 µg of DNA construct, 3 µL of JetPRIME, and the appropriate amount of JetPRIME buffer to create a 100-µL solution. Jeg3 cells previously seeded at a density of 1 × 10^6^ in 6-well culture plates had the medium changed to prewarmed (37°C) MEM without serum and were transfected by adding 100 µL of the above solution. After 4-hour incubations at 37°C, the medium was replaced with MEM containing serum.

### siRNA KDs

Cells were seeded as described elsewhere, in 24-well plates at a density of 25 000, and the medium was replaced on the day of the KD. The siRNA solution was prepared by combining 100 µL of cold (4°C) serum-free medium, 0.6 µL of siRNA, and 4 µL of INTERFERin (Polyplus). After 4 minutes elapsed, the tubes containing the siRNA were inverted gently. After an additional 9 minutes, 100 µL of the solution was added to each well and incubated at 37°C for 96 hours.

### Bacterial Strains and Growth Conditions

*L. monocytogenes* strain EGD BUG 600 (a gift from Pascale Cossart) was grown in brain-heart infusion (BHI) broth or agar (BD sciences) at 37°C. GFP–*L. monocytogenes* EGD BUG 2539 (gift from Pascale Cossart) was grown in the same conditions but selected for using chloramphenicol (25 mg/mL).

### *L. monocytogenes* Infections

*L. monocytogenes* bacteria were grown on BHI plates overnight in a standing incubator at 37°C. A single colony was selected and grown in BHI broth overnight in a shaking incubator at 37°C. Overnight cultures were diluted in fresh BHI (1:2) and incubated in the same conditions until an optical density of 1.00 at 600 nm was reached. The bacteria (1 mL) were centrifuged at 9,600*g* (25°C) and then washed with PBS^−/−^. This washing was repeated once. The pelleted bacteria were resuspended in MEM/DMEM without serum (37°C), then diluted 1:1000. The diluted bacteria (300 µL) were added to culture plates containing host cells with serum-free medium. The well plates were centrifuged at 9,600*g* for 2 minutes and incubated at 37°C. At the 2 hours after infection, the infected cells in the culture plates were washed 3 times with PBS^−/−^ and then with medium containing 10% FBS and 50 µg/mL gentamycin. Infected cells were incubated for ≥8 hours to allow cell-to-cell spreading to occur.

### *L. monocytogenes* Cell-to-Cell Spreading Assays

One batch of Jeg3 or HeLa cells were seeded at a density of 1 × 10^6^ in 6-well culture plates with medium and incubated at 37°C. The following day, these cells were infected as described above. At 2 hours after infection, the infected cells were washed 3 times with PBS^−/−^ and then medium containing 10% FBS, CellTracker Blue (1:50 dilution), and 50 µg/mL gentamycin. At 3 hours after infection, the infected and previously transfected/knocked-down cells were lifted using trypsin and counted. The 2 population of cells were combined at a ratio of 1:10 (ratio of infected to transfected/knocked-down cells) in 24-well plates containing glass coverslips and centrifuged at 214*g* for 3 minutes. After 6 hours of incubation at 37°C, the cells were washed 3 times with prewarmed (37°C) PBS^−/−^ and fixed with 3% paraformaldehyde (prepared in 150 mmol/L sodium chloride, 4 mmol/L sodium/potassium phosphate, and 5.0 mmol/L potassium chloride; pH 7.3) for 15 minutes. The cells were then washed with prewarmed PBS^−/−^ and permeabilized with 0.2% Triton X-100 in PBS^−/−^ for 5 minutes each. The cells were washed with prewarmed PBS^−/−^ 3 times for 10 minutes, and then filamentous actin was stained using Alexa Fluor 594–conjugated phalloidin prepared in PBS^−/−^ for 15 minutes. The cells were washed a final 3 times with prewarmed PBS^−/−^ and mounted onto glass microscope slides, using Prolong Diamond antifade mounting medium (with or without 4',6-diamidino-2-phenylindole [DAPI]).

### Immunolocalization

Infected cells on glass coverslips were washed 3 times with prewarmed (37°C) PBS^−/−^ and fixed with 3% paraformaldehyde for 15 minutes. After the sample were washed the samples for 5 minutes with PBS^−/−^, the cells were permeabilized using 0.2% Triton X-100 in PBS^−/−^ for 5 minutes. Following permeabilization, the cells were washed 3 times with PBS^−/−^, for 10 minutes each wash. All samples were blocked with 5% normal goat serum prepared in Tween-20 PBS (TPBS)/bovine serum albumin (BSA) (PBS^−/−^, 0.5% Tween 20, 0.1% BSA) for 20 minutes. The samples were incubated with primary antibodies prepared in TPBS/BSA overnight at 4°C. The following day, the samples were washed 3 times with TPBS/BSA for 10 minutes and incubated with Alexa Fluor 488– or Alexa Fluor 594–conjugated secondary antibodies at room temperature in the dark for 2 hours. Samples were washed 3 times with TPBS/BSA for 10 minutes and treated with Alexa Fluor 594–conjugated phalloidin prepared with TPBS/BSA for 20 minutes at room temperature in the dark. The samples were then washed 3 times with PBS^−/−^ and mounted onto glass microscope slides with Prolong Diamond antifade mounting medium (with DAPI).

### Threshold Analysis

HeLa, HeLa caveolin-1 KD and HeLa caveolin-1 KD/siRNA flotillin-1 cells were seeded and used for cell-to-cell spreading assays with wild-type (WT) HeLa cells infected with GFP*–L. monocytogenes,* as described above. Image acquisition and analysis was acquired through MetaMorph software. Images containing only the green channel were thresholded with the “exclusive state” setting in MetaMorph software. This enabled a distinction to be made between the objects measured (GFP–*L. monocytogenes*) and the rest of the image based on pixel intensity values. The exclusive state measures all gray-scale values equal to or outside the upper and lower limits of the GFP–*L. monocytogenes* channel. The infection foci were outlined using the “close” morphology filter with a circular filter shape (diameter, 50 pixels) to create a mask of the spreading bacteria. This mask was measured using “image morphology analysis” to provide comparable values among the 3 experimental conditions.

### Western Blot and Lysate Preparation

Cells were washed 3 times with prewarmed PBS^−/−^ and then treated with RIPA lysis buffer (150 mmol/L sodium chloride, 50 mmol/L Tris [pH 7.4], 5 mmol/L EDTA, 1% Nonidet P-40, 1% deoxycholic acid, and 10% sodium dodecyl sulfate [SDS]) containing a cOmplete Mini EDTA-free protease inhibitor cocktail (Roche) on ice for 5 minutes. Cells scrapers were used to scrape the cells, and the lysate was collected into a 1.7-mL microcentrifuge tube. The samples were centrifuged for 10 minutes at 9,600*g* to pellet the debris. The supernatant was collected into fresh tubes. Liquid nitrogen was used to flash freeze the lysate for storage at −80°C. Protein concentrations were measured using a bicinchoninic acid assay kit (Pierce). Lysates were prepared with 6× sample buffer, boiled (100°C) for 10 minutes and subsequently vortexed at maximum speed for 1 minute. Equal amounts of protein were loaded onto 10% SDS-polyacrylamide gels and resolved by electrophoresis. The proteins in the gels were transferred onto nitrocellulose membrane using a Trans Blot SD semidry transfer cell (Bio-Rad) for 45 minutes at 75 mA.

The membranes were washed for 5 minutes in TBST (Tris-buffered saline, 0.05% Tween 20) and blocked for 1 hour with 4% Blotto milk (Bio-Rad) prepared in TBST shaken at room temperature. The membranes were stained overnight with primary antibodies diluted with TBST + 1% BSA at 4°C. The next day, the membranes were washed 3 times for 10 minutes each with TBST and subjected to secondary antibodies (horseradish peroxidase–conjugated goat anti-rabbit or anti-mouse antibodies). Membranes were treated with Western Lightning Plus-ECL (PerkinElmer) and imaged on a Fujifilm LAS-4000 imager. To confirm equivalent protein levels, membranes were stripped using a mild stripping buffer (1.5% glycine, 0.1% SDS, and 1% Tween 20; pH 2.2), and the same steps were repeated to reprobe the membranes using rabbit anti–flotillin-2 and mouse anti–α-tubulin targeting antibodies (Developmental Studies Hybridoma Bank; 12G10, Sigma-Aldrich; 1:500 dilution for Western blotting).

### Microscopy

A Leica DMI4000B inverted fluorescent microscope was used to view all slides. Photos of the samples were acquired using a Hamamatsu Orca R2 CCD camera. All devices were controlled using MetaMorph Imaging System software, and images were analyzed and edited with Metamorph Imaging System or ImageJ/Fiji software.

### Statistical Analysis

All results involving immunofluorescence microscopy were performed ≥3 times (n = 3) using ≥40 different fields of view for analysis. The experimental variables were normalized to the controls. Statistical tests were either 2-tailed *t* tests or 1-way analysis of variance with Tukey test for statistically significant values with a *P* value less than <.01.

## RESULTS

**Figure 1.**
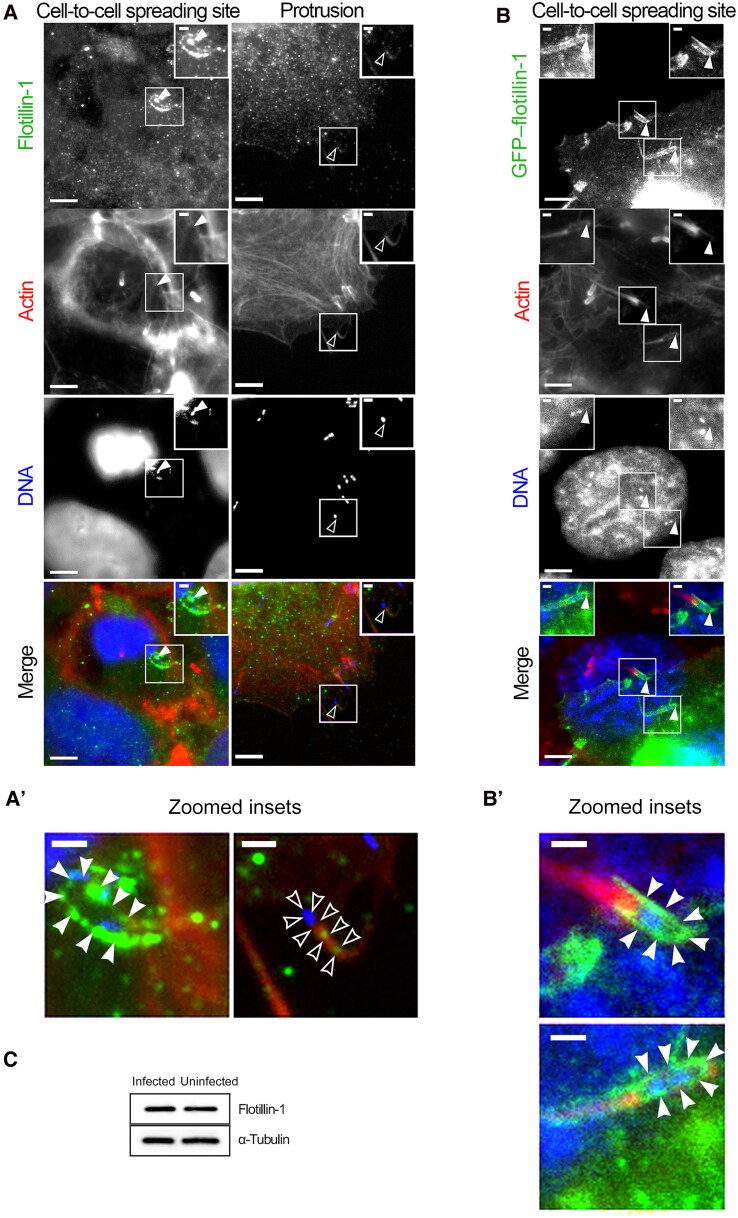
Localization of flotillin-1 during *Listeria monocytogenes* infections. *A*. Cells infected with *L. monocytogenes* for 8 hours were fixed and stained with rabbit polyclonal flotillin-1 targeting antibodies, Alexa Fluor 594 phalloidin to visualize F-actin, and 4',6-diamidino-2-phenylindole [DAPI] to stain for host and bacterial DNA. Boxed regions illustrate spreading events wherein flotillin was localized to invaginations at cell-to-cell spreading sites but not to protrusions extending into open space. *B*. Jeg3 cells transfected with green fluorescent protein (GFP)­–flotillin-1 were combined wild-type Jeg3 cells previously infected with *L. monocytogenes.* After 9 hours of total infection the cells were fixed and stained with Alexa Fluor 594 to visualize F-actin and DAPI to visualize host and bacterial DNA showing GFP–flotillin-1 localization specifically at the invaginations. *A’, B’,* Zoomed insets of boxed regions. *C,* Whole Jeg3 cell lysates from uninfected and infected cells were probed for endogenous flotillin-1. α-Tubulin is shown as a loading control. Scale bars represent 5 µm in the zoomed-out images and 2.25 µm in the zoomed insets; open arrowheads, *L. monocytogenes* protrusions; and closed arrowheads, invaginations.

Caveolin-mediated endocytosis is the only endocytic mechanism that has been identified to function in *L. monocytogenes* cell-to-cell spreading at receiving cell invaginations [15, 16]. Although this finding is important for the cell-to-cell movement of the bacteria, it accounted for only approximately 70% of bacterial spreading in recipient cells with caveolin-1 KD. To investigate other strategies that might account for the remaining cell-to-cell transfer of *L. monocytogenes* we examined the involvement of flotillin-mediated endocytosis. We initially immunolocalized endogenous flotillin-1 during cultured cell infections and found it concentrated as puncta at sites of cell-to-cell spreading (Figure 1*A*). To determine whether the localization of flotillin-1 labeled the invaginations or protrusions (listeriopods), we examined *L. monocytogenes* protrusions in loosely seeded samples where the protrusions could extend into open space, and we found flotillin-1 absent at those sites (Figure 1*A* and Figure 1*A'*). This suggested that flotillin-1 was likely concentrated at the invaginations. To confirm this, we used cell-to-cell spreading assays in which infected Jeg3 cells were combined with uninfected, GFP-tagged flotillin-1 transfected “receiving” cells. Here, we found GFP–flotillin-1 localized along the entire length of the invaginations in the receiving cells of the infections (Figure 1*B, B’*). The accumulation of flotillin-1 at invaginations did not result in any obvious change to the endogenous flotillin-1 levels compared with uninfected Jeg3 samples (Figure 1*C*).

**Figure 2.**
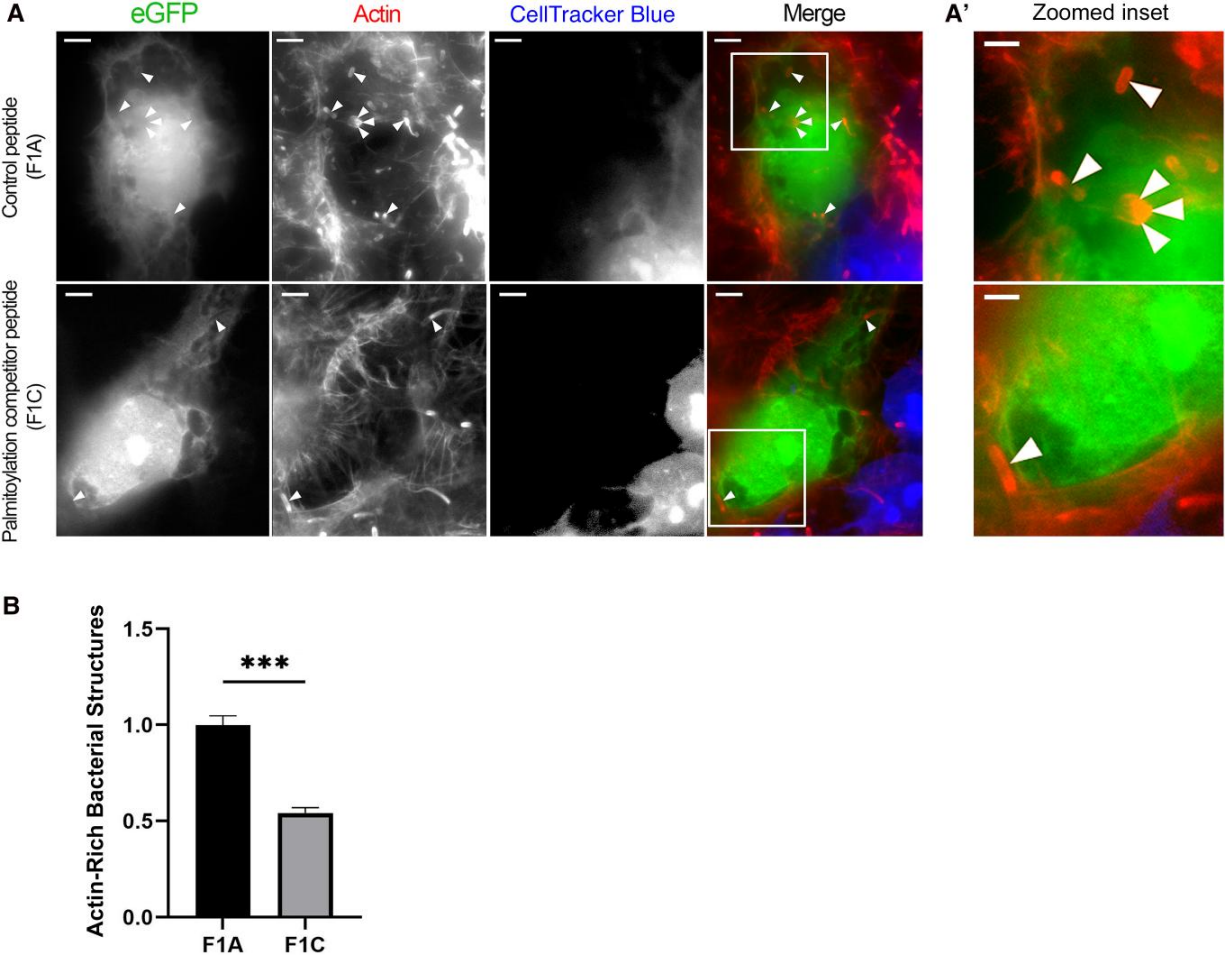
Cell-to-cell spreading assays using constructs in competition with endogenous flotillin-1 for palmitoylation. *A,* Control (F1A) or competitive inhibiting (F1C) peptides used in cell-to-cell spreading assays. CellTracker Blue–stained cells that were initially infected with *Listeria monocytogenes* (sending cells) were combined with uninfected Jeg3 cells transfected with tetracycline reponse element-enchanced green fluorescent protein (TRE-eGFP)-F1A/F1C constructs (receiving cells). Following 6-hour incubations, the cells were fixed and stained with Alexa Fluor 594 phalloidin. Arrowheads point to actin-rich structures counted as a measure of cell-to-cell spreading. Scale bar represents 5 µm. *A’,* Zoomed insets of boxed regions for the control peptide (*top*) and the palmitoylation competitive peptide (*bottom*) highlight differences in the abundance of actin-rich structures. The red channel was enhanced to ease the visualization of actin. Scale bar represents 2.25 µm. *B,* Actin-rich structures were counted in ≥50 receiving cells of the cell-to-cell spreading assays in 3 separate experiments. Competitive inhibiting peptide counts were normalized to the wild type. A 2-tailed *t* test with Welch correction was used to analyze the data, resulting in significantly fewer actin-rich structures in the F1C than in the F1A assays. ***P < .001.

To investigate how flotillin-1 could influence *L. monocytogenes* moving from one cell to another, we preformed cell-to-cell spreading assays in which preinfected CellTracker Blue–stained cells were combined with uninfected cells transfected with eGFP-peptide constructs that competed with endogenous flotillin for palmitoylation (F1C). Palmitoylation is a posttranslational modification required for flotillin-1 to bind with distinct membrane domains. A control peptide (F1A) was used for comparison. As a measure of cell-to-cell spreading, the actin-rich structures generated during the *L. monocytogenes* lifecycle (actin clouds, comet tails, and listeriopods) were enumerated in only the transfected (receiving) cells of the experiments. We found that there was a significant decrease (about 50%) in the cells that expressed the competitive peptide, which functioned to inhibit endogenous flotillin-1 from binding to the membrane, compared with the control peptide (Figure 2*B*).

**Figure 3.**
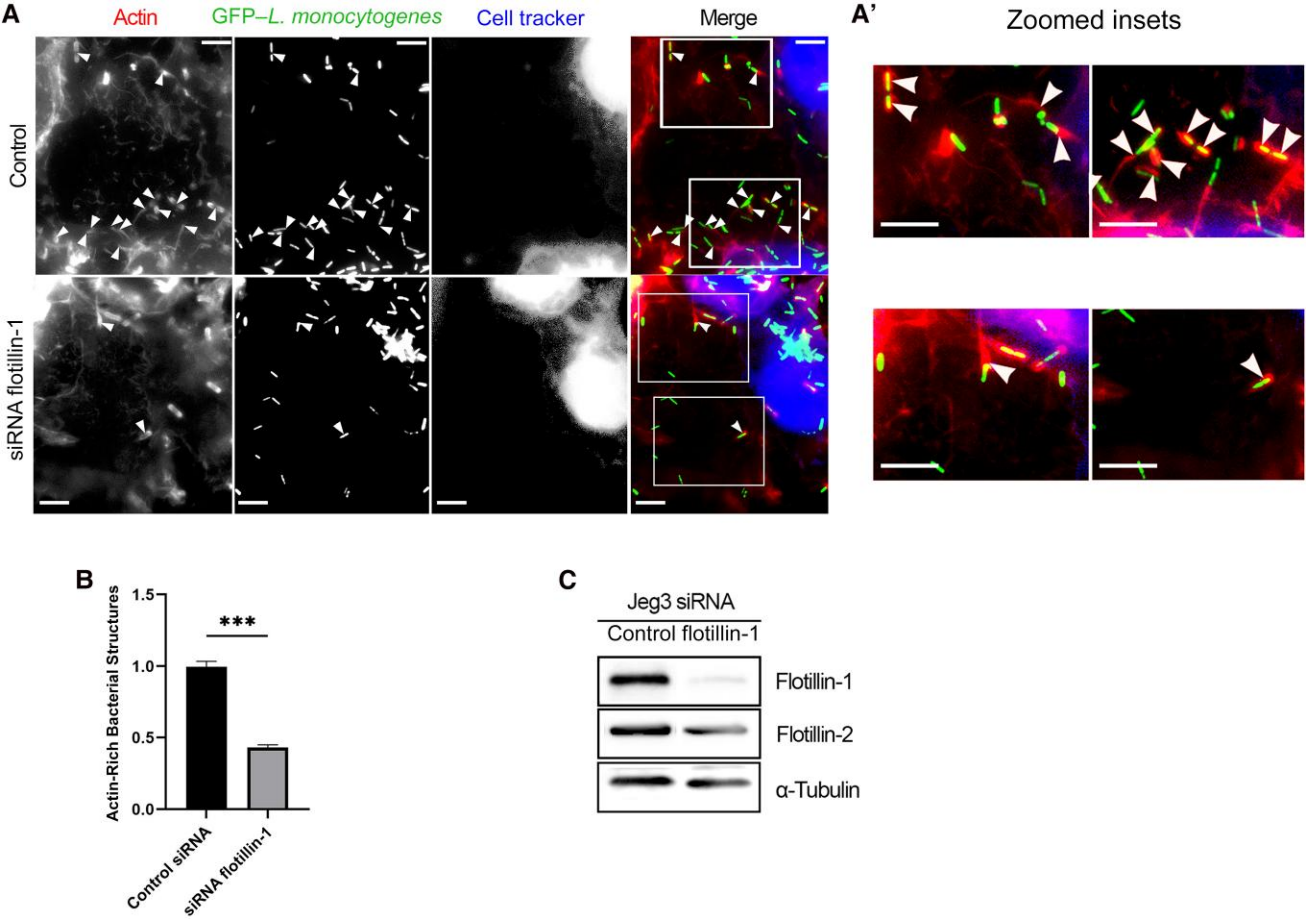
The effects of small interfering RNA (siRNA) flotillin-1 knockdown (KD) in Jeg3 cells on the spreading events of *Listeria monocytogenes. A,* Control Jeg3 cells exposed to scrambled nontargeting siRNA (control) or siRNA flotillin-1 were subjected to cell-to-cell spreading assays, as described elsewhere. Following 6-hour incubations, the cells were fixed and stained with Alexa Fluor 594 phalloidin. The actin-rich structures were enumerated as a measure of cell-to-cell spreading (some of the actin-rich structures are indicated by arrowheads). Scale bar represents 5 µm. Abbreviation: GFP, green fluorescent protein. *A’,* Zoomed insets of boxed regions for control (*top*) and siRNA flotillin-1 (*bottom*) illustrating differences in the abundance of actin-rich structures. Scale bar represents 5 µm. *B,* Actin-rich structures were counted in ≥50 receiving cells of the mixed cell assays in 3 independent experiments with the siRNA counts normalized to the scramble controls. A 2-tailed *t* test with Welch correction was used to analyze the data, which resulted in significantly fewer actin-rich structures in the siRNA assays compared with control siRNA. ***P < .001. *C,* Western blot showing the efficacy of the flotillin-1 KD compared with controls. α-Tubulin was used as a loading control. Endogenous flotillin-2 expression levels were also probed for during flotillin-1 KD.

To further characterize the potential role of flotillin-1 during *L. monocytogenes* movement from one cell to another, we again performed cell-to-cell spreading assays but used siRNA-mediated KDs of flotillin-1 in the receiving Jeg3 cells. To do this, cells previously infected with GFP–*L. monocytogenes* were combined with uninfected flotillin-1 KD cells, and enumeration of the actin-rich structures in the KD cells was again used as a measure of cell-to-cell spreading (Figure 3*A*). In a similar fashion as the flotillin-1 palmitoylation peptide experiment, we found a decrease of about 50% in the number of the actin-rich structures generated in the flotillin-1 KD cells, compared with the control siRNA-treated cells (Figure 3*B*). Western blot analysis showed reduction in flotillin-1 protein levels in the Jeg3 cells (Figure 3*C*). The blots were stripped and reprobed with antibodies against α-tubulin (loading control) and flotillin-2 to determine whether the flotillin-1 KD influenced flotilli-2 levels. A slightly lower expression level of flotillin-2 in the cells with flotillin-1 KD was observed, which aligns with findings of a previous study showing that knocking down flotillin-1 slightly decreased flotillin-2 levels [26].

**Figure 4.**
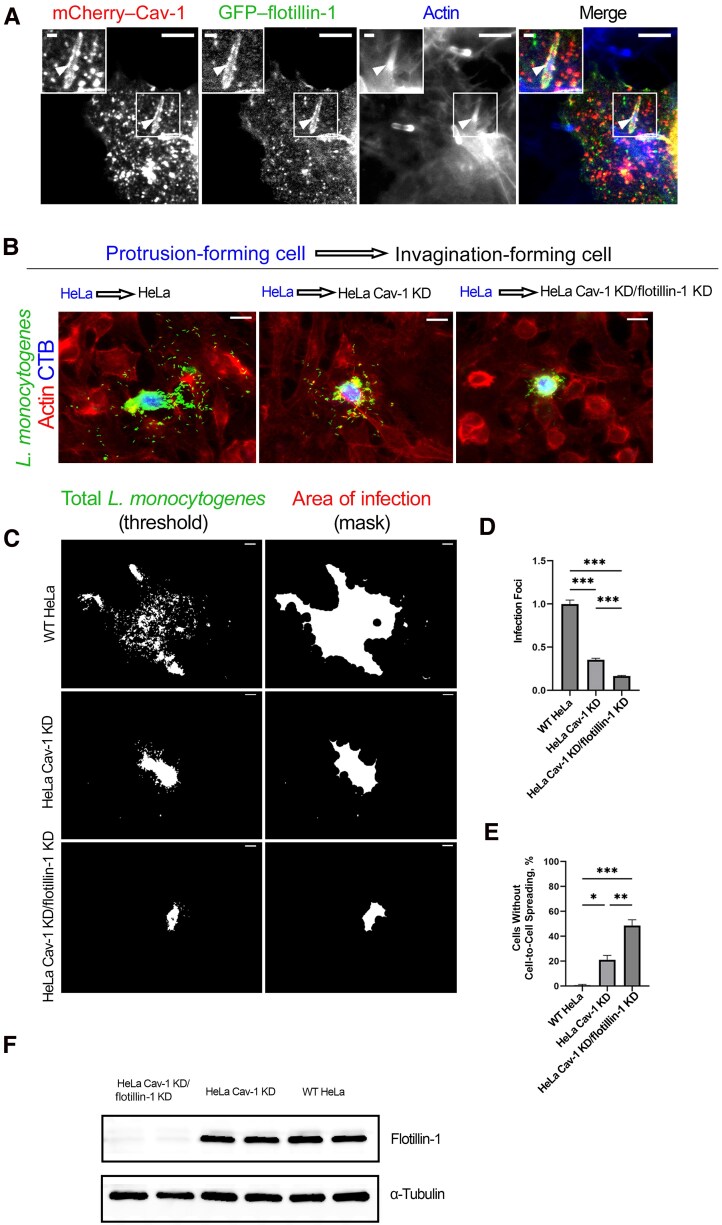
Flotillin-1 further hinders *Listeria monocytogenes* cell-to-cell spreading in cells devoid of caveolin-1 (Cav-1). *A,* Colocalization of green fluorescent protein (GFP)–flotillin-1 and mCherry–Cav-1 at invaginations were analyzed in cells infected with *L. monocytogenes*. Cells were also stained with Alexa Fluor 405 phalloidin to label F-actin. Arrowheads indicate invagination sites. Scale bars represent 5 µm in the zoomed-out images and 2.25 µm in the zoomed insets. *B,* Cell-to-cell spreading events between the 3 experimental conditions where wild-type (WT) CellTracker Blue­–stained (CTB) HeLa cells previously infected with GFP–*L. monocytogenes* were combined with HeLa, HeLa Cav-1 knockdown (KD) or HeLa Cav-1 KD small interfering RNA (siRNA) flotillin-1 cells for 6 hours. Cells were fixed, and F-actin was stained with Alexa Fluor 594 phalloidin. Scale bar represents 20 µm. *C,* Images of the infection foci measured among the 3 experimental conditions. GFP–*L. monocytogenes* was thresholded (*left*) and the infection foci masked (*right*) using Metamorph software to visualize the cell-to-cell spreading events. *D,* Quantification of the infection foci of HeLa, HeLa Cav-1 KD and HeLa Cav-1 KD siRNA flotillin-1 cells. At least 40 cells were measured in each experimental condition from 3 independent replicates. The HeLa Cav-1 KD (30.5% of the WT area) and HeLa Cav-1 KD siRNA flotillin-1 (16% of the WT area) values were normalized to WT HeLa spreading events and compared using 1-way analysis of variance (ANOVA) with Tukey test. ***P < .001. Scale bar represents 20 µm. *E,* Enumeration of cells in which bacteria were restricted to the initially infected HeLa cell to illustrate unsuccessful spreading. At least 40 cells were observed from 3 independent replicates in each experimental condition and compared using 1-way ANOVA with Tukey test. WT HeLa cells had 0% blocked cell-to-cell spreading, while an average of 20% of the HeLa Cav-1 KD cells and 40% of the HeLa Cav-1 KD siRNA flotillin-1 cells had blocked spreading events. *P < .05; **P < .01; ***P < .001. *F,* Whole-cell lysates from HeLa Cav-1 KD/flotillin-1 KD and HeLa Cav-1 KD (scrambled nontargeting siRNA), and HeLa cells were collected and probed for endogenous flotillin-1 using rabbit polyclonal antibodies. α-Tubulin was used as a loading control.

We next set out to determine the functional importance of flotillin-1 in conjunction with caveolin-1–mediated endocytosis at *L. monocytogenes* invaginations. Previous research has shown that *L. monocytogenes* uses caveolar endocytic components for internalization into neighboring cells [15, 16]. To determine whether flotillin-1 localized to the same invagination sites as caveolin-1, we performed cell-to-cell spreading assays using cells cotransfected with GFP–flotillin-1 and mCherry–caveolin-1. We found that both proteins localized to the same *L. monocytogenes*–containing invaginations (Figure 4*A*). However, despite their localization at the structures, they did not always colocalize to the same puncta. Cell-to-cell spreading areas of infection was previously analyzed using HeLa caveolin-1 KD cells, but spreading was not eliminated, as approximately 30% of the area of infection on the coverslip monolayers remained [16].

To test whether flotillin-1 contributed to this remaining bacterial spreading, we replicated the caveolin-1 KD cell-to-cell spreading assays while also knocking down flotillin-1 in those cells, thus using HeLa caveolin-1 KD/flotillin-1 KD cells. Western blot analysis indicated a dramatic reduction in flotillin-1 protein levels in the caveolin-1 KD/flotillin-1 KD cells (Figure 4*F*). When we examined the monolayers of cells infected with GFP–*L. monocytogenes* that were stained for F-actin, we found a significant decrease in the area of bacterial spreading among the 3 cell lines (Figure 4*B*). The HeLa caveolin-1 KD cell cell-to-cell spreading assays showed a reduction of 69.5% compared with the spreading in WT HeLa cells, comparable to what Dhanda et al reported [16], while the HeLa caveolin-1 KD/flotillin-1 KD cells had a total reduction of approximately 85% compared with WT HeLa cell *L. monocytogenes* spreading (Figure 4*C* and 4*D*). As the area of a single cell could account for much of the remaining 15% of area, we also enumerated the number of cells that had no spreading (with all of the bacteria contained within a single cell) and found that the HeLa caveolin-1 KD group had an average of 21.15% of bacteria restricted to the original host cell, whereas the caveolin-1 KD/flotillin-1 KD group had average of 48.50% of cells with completely restricted spreading (Figure 4*E*).

## DISCUSSION

Efforts to understand the molecular mechanisms that govern *L. monocytogenes* cell-to-cell spreading have largely focused on protrusion formation in the initially infected host cells. However, growing interest in the endocytic strategies exploited by microbes during their cell-to-cell transfer have emerged in the past number of years [27–29]. Caveolin-mediated endocytosis was shown to be hijacked by *L. monocytogenes* for cell-to-cell spreading [15, 16, 30]. One study achieved a 95% depletion of caveolin-1, but approximately 30% of *L. monocytogenes* cell-to-cell spreading remained. While the residual caveolin-1 may have been enough for spreading to occur, we set out to explore whether other endocytic mechanisms may be involved during *L. monocytogenes* cell-to-cell transfer to account for the remaining spreading still seen in the caveolin-1 KD cells [16].

Flotillin-1 is a lipid raft membrane–associated protein that has roles in endocytosis [21 , 31]. In the current study we demonstrated how flotillin-1 is involved with listeriopods being internalized into neighboring cells and that the down-regulation of this protein alone inhibited intracellular dissemination. A further reduction in spreading was evident in cells with both flotillin-1 and caveolin-1 KD, was evident and nearly 50% of *L. monocytogenes* infected cells were completely blocked from dissemination out of their initially infected cells. The remaining host cell-to-cell transfer of *L. monocytogenes* could be due to the properties of KDs over knockouts, as there are still vestigial caveolin-1/flotillin-1 proteins present.

While few studies have explored the involvement of flotillins with pathogens, one examined the invasion of *Pseudomonas aeruginosa* and demonstrated that it exploits flotillin-1 for its internalization [32]. Our study extends this by showing *L. monocytogenes* uses flotillin-1 not for growth, invasion or vacuole development but to mediate the internalization of actin-rich protrusions that extend tens of micrometers during cell-to-cell spreading [33–36]. To our knowledge, these are the largest structures that have been shown to use flotillin-based endocytosis for intracellular spread to date.

There is controversy in the field as to whether flotillins function independently or cooperate with caveolin on the same vesicles [22, 23, 37–40]. When we performed cell-to-cell spreading assays in Jeg3 cells simultaneously expressing GFP–flotillin-1 and mCherry–caveolin-1 in the receiving cells of the infection, we found that both flotillin-1 and caveolin-1 localized at the invaginations during these endocytic events. However, the localization was mismatched along the sites at these large structures. This indicated that while both proteins were present at the invaginations, they could be functioning at distinct areas within the structure. The work by Frick et al [38] supports this, as they found that flotillins and caveolin-1 occupy topographically distinct areas of the plasma membrane. Consequently, the synergistic or independent activities of flotillin-1 and caveolin-1 during classic (nanometer-sized) and large (micrometer-sized) endocytosis will require additional examination to determine whether flotillins work independently or cooperatively with caveolin-1 at subdomains of the membrane.

Flotillin and caveolin both localize to lipids rafts, which are enriched in cholesterol and sphingolipids. Although these lipids have not been directly linked to *L. monocytogenes* cell-to-cell spreading, previous studies indicated that depletion of cholesterol disrupts caveolae and inhibits the flotillin-mediated endocytosis of proteins [37, 41]. Thus, lipid depletions may impair *L. monocytogenes* spread, though this also requires further investigation.

**Figure 5.**
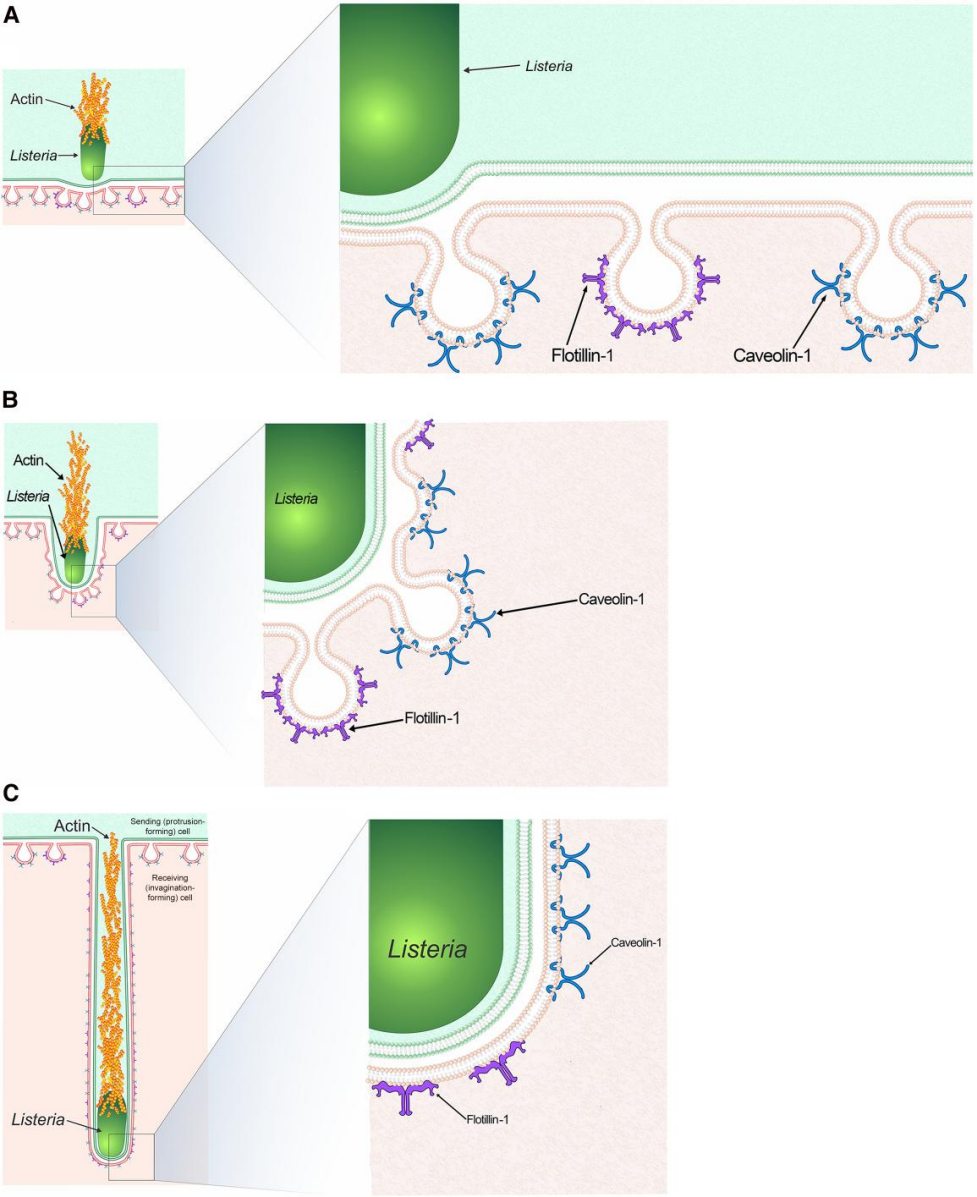
Hypothesized model of the endocytic mechanisms responsible for *Listeria monocytogenes* cell-to-cell spreading. *A, L. monocytogenes* initially binds to its target neighboring cell (recipient cell) for an invagination to begin forming, where different flotillin-1 pits and caveolin-1–rich caveolae are present. *B,* As *L. monocytogenes* begins to spread into the recipient cell, the invagination deepens, the flotillin-1 pits and caveolae begin to flatten with the proteins still intact within the plasma membrane. *C,* Flotillin-1 pits and caveolae fully flatten as the *L. monocytogenes–*containing invagination fully develops, while the flotillin-1 and caveolar proteins remain localized to the membrane.

Past studies have found that caveolae flatten in response to mechanical stress of the cell [42]. Thus, we propose an updated model (modified from [16]) in which *L. monocytogenes* protrusions bind to the plasma membrane where both flotillin-1– and caveolin-1–rich endocytic pits are present. Those structures then flatten as the invagination elongates (Figure 5). Our work provides evidence that flotillins provide an additional endocytic mechanism that *L. monocytogenes* co-opt for cell-to-cell transfer and shows that flotillins likely have the ability to work with caveolin to internalize very large cellular structures.

## References

[1] Centers for Disease Control and Prevention . 2024. https://www.cdc.gov/listeria/hcp/clinical-overview/index.html. Accessed October 2024.

[2] Lecuit M . Understanding how *Listeria monocytogenes* targets and crosses host barriers. Clin Microbiol Infect 2005; 11:430–6. 15882192 10.1111/j.1469-0691.2005.01146.x

[3] Dramsi S, Biswas I, Maguin E, Braun L, Mastroeni P, Cossart P. Entry of *L. monocytogenes* into hepatocytes requires expression of inIB, a surface protein of the internalin multigene family. Mol Microbiol 1995; 16:251–61. 7565087 10.1111/j.1365-2958.1995.tb02297.x

[4] Gaillard JL, Berche P, Frehel C, Gouin E, Cossart P. Entry of *L. monocytogenes* into cells is mediated by internalin, a repeat protein reminiscent of surface antigens from gram-positive cocci. Cell 1991; 65:1127–41. 1905979 10.1016/0092-8674(91)90009-n

[5] Veiga E, Guttman JA, Bonazzi M, et al Invasive and adherent bacterial pathogens co-opt host clathrin for infection. Cell Host Microbe 2007; 2:340–51. 18005755 10.1016/j.chom.2007.10.001 PMC2803069

[6] Veiga E, Cossart P. *L. monocytogenes* hijacks the clathrin-dependent endocytic machinery to invade mammalian cells. Nat Cell Biol 2007; 7:894–900. 10.1038/ncb1292 16113677

[7] Geoffroy C, Gaillard JL, Alouf JE, Berche P. Purification, characterization, and toxicity of the sulfhydryl-activated hemolysin listeriolysin O from *L. monocytogenes*. Infect Immun 1987; 55:1641–6. 3110067 10.1128/iai.55.7.1641-1646.1987 PMC260571

[8] Welch MD, Rosenblatt J, Skoble J, Portnoy DA, Mitchison TJ. Interaction of human Arp2/3 complex and the *L. monocytogenes* ActA protein in actin filament nucleation. Science 1998; 281:105–8. 9651243 10.1126/science.281.5373.105

[9] Welch M, Iwamatsu A, Mitchison T. Actin polymerization is induced by Arp 2/3 protein complex at the surface of *L. monocytogenes*. Nature 1997; 385:265–9. 9000076 10.1038/385265a0

[10] Kocks C, Gouin E, Tabouret M, Berche P, Ohayon H, Cossart P. *L. monocytogenes*-induced actin assembly requires the actA gene product, a surface protein. Cell 1992; 68:521–31. 1739966 10.1016/0092-8674(92)90188-i

[11] Sechi AS, Wehland J, Small JV. The isolated comet tail pseudopodium of *L. monocytogenes:* a tail of two actin filament populations, long and axial and short and random. J Cell Biol 1997; 137:155–67. 9105044 10.1083/jcb.137.1.155 PMC2139863

[12] Cameron LA, Svitkina TM, Vignjevic D, Theriot JA, Borisy GG. Dendritic organization of actin comet tails. Curr Biol 2001; 11:130–5. 11231131 10.1016/s0960-9822(01)00022-7

[13] Jasnin M, Asano S, Gouin E, et al Three-dimensional architecture of actin filaments in *L. monocytogenes* comet tails. Proc Natl Acad Sci U S A 2013; 110:20521–6. 24306931 10.1073/pnas.1320155110 PMC3870744

[14] Tilney LG, Portnoy DA. Actin filaments and the growth, movement, and spread of the intracellular bacterial parasite, *L. monocytogenes*. J Cell Biol 1989; 109:1597–608. 2507553 10.1083/jcb.109.4.1597 PMC2115783

[15] Sanderlin AG, Vondrak C, Scricco AJ, Fedrigo I, Ahyong V, Lamason RL. RNAi screen reveals a role for PACSIN2 and caveolins during bacterial cell-to-cell spread. Mol Biol Cell 2019; 30:2124–33. 31242077 10.1091/mbc.E19-04-0197 PMC6743452

[16] Dhanda AS, Yu C, Lulic KT, et al *L. monocytogenes* exploits host caveolin for cell-to-cell spreading. mBio 2020; 11:e02857-19. 31964732 10.1128/mBio.02857-19 PMC6974566

[17] Ireton K . Molecular mechanisms of cell-cell spread of intracellular bacterial pathogens. Open Biol 2013; 3:130079. 23864553 10.1098/rsob.130079 PMC3728924

[18] Rivera-Milla E, Stuermer CA, Málaga-Trillo E. Ancient origin of reggie (flotillin), reggie-like, and other lipid-raft proteins: convergent evolution of the SPFH domain. Cell Mol Life Sci 2006; 63:343–57. 16389450 10.1007/s00018-005-5434-3 PMC11135987

[19] Morrow IC, Rea S, Martin S, et al Flotillin-1/reggie-2 traffics to surface raft domains via a novel Golgi-independent pathway: identification of a novel membrane targeting domain and a role for palmitoylation. J Biol Chem 2002; 277:48834–41. 12370178 10.1074/jbc.M209082200

[20] Neumann-Giesen C, Falkenbach B, Beicht P, et al Membrane and raft association of reggie-1/flotillin-2: role of myristoylation, palmitoylation and oligomerization and induction of filopodia by overexpression. Biochem J 2004; 378:509–18. 14599293 10.1042/BJ20031100 PMC1223955

[21] Prohaska R, Salzer U. Flotillin-1 (*flot1*). In: Choi S, ed. Encyclopedia of signaling molecules. New York: Springer, 2012:624–630.

[22] Aït-Slimane T, Galmes R, Trugnan G, Maurice M. Basolateral internalization of GPI-anchored proteins occurs via a clathrin-independent flotillin-dependent pathway in polarized hepatic cells. Mol Biol Cell 2009; 20:3792–800. 19605558 10.1091/mbc.E09-04-0275 PMC2735478

[23] Glebov OO, Bright NA, Nichols BJ. Flotillin-1 defines a clathrin-independent endocytic pathway in mammalian cells. Nat Cell Biol 2006; 8:46–54. 16341206 10.1038/ncb1342

[24] Vercauteren D, Piest M, van der Aa LJ, et al Flotillin-dependent endocytosis and a phagocytosis-like mechanism for cellular internalization of disulfide-based poly(amido amine)/DNA polyplexes. Biomaterials 2011; 32:3072–84. 21262529 10.1016/j.biomaterials.2010.12.045

[25] Cremona ML, Matthies HJ, Pau K, et al Flotillin-1 is essential for PKC-triggered endocytosis and membrane microdomain localization of DAT. Nat Neurosci 2011; 14:469–77. 21399631 10.1038/nn.2781 PMC3066276

[26] Amaddii M, Meister M, Banning A, et al Flotillin-1/reggie-2 protein plays dual role in activation of receptor-tyrosine kinase/mitogen-activated protein kinase signaling. J Biol Chem 2012; 287:7265–78. 22232557 10.1074/jbc.M111.287599 PMC3293549

[27] Vondrak CJ, Sit B, Suwanbongkot C, Macaluso KR, Lamason RL. A conserved interaction between the effector Sca4 and host endocytic machinery suggests additional roles for Sca4 during rickettsial infection. bioRxiv [Preprint: not peer reviewed]. 24 June 2024. Available from: 10.1101/2024.06.24.600492. PMC11629629 39535192

[28] Heinzen RA, Scidmore MA, Rockey DD, Hackstadt T. Differential interaction with endocytic and exocytic pathways distinguish parasitophorous vacuoles of *Coxiella burnetii* and *Chlamydia trachomatis*. Infect Immun 1999; 64:796–809. 10.1128/iai.64.3.796-809.1996 PMC173840 8641784

[29] Fukumatsu M, Ogawa M, Arakawa S, et al *Shigella* targets epithelial tricellular junctions and uses a noncanonical clathrin-dependent endocytic pathway to spread between cells. Cell Host Microbe 2012; 11:325–36. 22520461 10.1016/j.chom.2012.03.001

[30] Radhakrishnan P, Sathe M, Theriot JA. *Listeria monocytogenes* co-opts caveolin-mediated E-cadherin trafficking and macropinocytosis for epithelial cell-to-cell spread. bioRxiv [Preprint: not peer reviewed]. 6 April 2022. Available from: 10.1101/2022.04.06.487361.

[31] Hoehne M, de Couet HG, Stuermer CA, Fischbach KF. Loss- and gain-of-function analysis of the lipid raft proteins Reggie/Flotillin in *Drosophila*: they are post translationally regulated, and misexpression interferes with wing and eye development. Mol Cell Neurosci 2005; 30:326–38. 16154361 10.1016/j.mcn.2005.07.007

[32] Brandel A, Aigal S, Lagies S, et al The Gb3-enriched CD59/flotillin plasma membrane domain regulates host cell invasion by *Pseudomonas aeruginosa*. Cell Mol Life Sci 2021; 78:3637–56. 33555391 10.1007/s00018-021-03766-1 PMC8038999

[33] Babuke T, Tikkanen R. Dissecting the molecular function of reggie/flotillin proteins. Eur J Cell Biol 2007; 86:525–32. 17482313 10.1016/j.ejcb.2007.03.003

[34] Korhonen JT, Puolakkainen M, Haveri A, et al Intracellular survival of *Chlamydia pneumoniae* is dependent on flotillin-1-mediated cholesterol transport. Traffic 2012; 13:1110–21.

[35] Xiong Q, Lin M, Huang W, Rikihisa Y. Infection by *Anaplasma phagocytophilum* requires recruitment of low-density lipoprotein cholesterol by flotillins. mBio 2019; 10:e02783-18. 30914515 10.1128/mBio.02783-18 PMC6437059

[36] Huang W, Xiong Q, Lin M, Rikihisa Y. *Anaplasma phagocytophilum* hijacks flotillin and NPC1 complex to acquire intracellular cholesterol for proliferation, which can be inhibited with ezetimibe. mBio 2021; 12:e0229921. 34544283 10.1128/mBio.02299-21 PMC8546544

[37] Schneider A, Rajendran L, Honsho M, et al Flotillin-dependent clustering of the amyloid precursor protein regulates its endocytosis and amyloidogenic processing in neurons. J Neurosci 2008; 28:2874–82. 18337418 10.1523/JNEUROSCI.5345-07.2008 PMC6670660

[38] Frick M, Bright NA, Riento K, Bray A, Merrified C, Nichols BJ. Coassembly of flotillins induces formation of membrane microdomains, membrane curvature, and vesicle budding. Curr Biol 2007; 17:1151–6. 17600709 10.1016/j.cub.2007.05.078

[39] Vassilieva EV, Ivanov AI, Nusrat A. Flotillin-1 stabilizes caveolin-1 in intestinal epithelial cells. Biochem Biophys Res Commun 2009; 379:460–5. 19121286 10.1016/j.bbrc.2008.12.118 PMC2867594

[40] Volonté D, Galbiati F, Li S, Nishiyama K, Okamoto T, Lisanti MP. Flotillins/cavatellins are differentially expressed in cells and tissues and form a hetero-oligomeric complex with caveolins in vivo: characterization and epitope-mapping of a novel flotillin-1 monoclonal antibody probe. J Biol Chem 1999; 274:12702–9. 10212252 10.1074/jbc.274.18.12702

[41] Dreja K, Voldstedlund M, Vinten J, Tranum-Jensen J, Hellstrand P, Swärd K. Cholesterol depletion disrupts caveolae and differentially impairs agonist-induced arterial contraction. Arterioscler Thromb Vasc Biol 2002; 22:1267–72. 12171786 10.1161/01.atv.0000023438.32585.a1

[42] Sinha B, Köster D, Ruez R, et al Cells respond to mechanical stress by rapid disassembly of caveolae. Cell 2011; 144:402–13. 21295700 10.1016/j.cell.2010.12.031 PMC3042189

